# Yellow Fever Outbreak, Imatong, Southern Sudan

**DOI:** 10.3201/eid1006.030738

**Published:** 2004-06

**Authors:** Clayton O. Onyango, Victor O. Ofula, Rosemary C. Sang, Samson L. Konongoi, Abdourahmane Sow, Kevin M. De Cock, Peter M. Tukei, Fredrick A. Okoth, Robert Swanepoel, Felicity J. Burt, Norman C. Waters, Rodney L. Coldren

**Affiliations:** * World Health Organization (WHO) Collaborating Center for Arbovirus and Viral Hemorrhagic Fever Reference and Research, Nairobi, Kenya;; † WHO South Sudan, Warwick Center, Nairobi, Kenya;; ‡ Centers for Disease Control and Prevention, Atlanta, Georgia, USA;; § Kenya Medical Research Institute, Nairobi, Kenya;; ¶ National Institute for Communicable Diseases, Johannesburg, Republic of South Africa;; # U.S. Army Medical Research Unit-Kenya, Nairobi, Kenya

**Keywords:** Yellow fever, ELISA, Oligonucleotide primer, amplicon, nucleotide sequence

## Abstract

In May 2003, the World Health Organization received reports about a possible outbreak of a hemorrhagic disease of unknown cause in the Imatong Mountains of southern Sudan. Laboratory investigations were conducted on 28 serum samples collected from patients in the Imatong region. Serum samples from 13 patients were positive for immunoglobulin M antibody to flavivirus, and serum samples from 5 patients were positive by reverse transcription–polymerase chain reaction with both the genus *Flavivirus*–reactive primers and yellow fever virus–specific primers. Nucleotide sequencing of the amplicons obtained with the genus *Flavivirus* oligonucleotide primers confirmed yellow fever virus as the etiologic agent. Isolation attempts in newborn mice and Vero cells from the samples yielded virus isolates from five patients. Rapid and accurate laboratory diagnosis enabled an interagency emergency task force to initiate a targeted vaccination campaign to control the outbreak.

Yellow fever virus (YFV), an arthropod-borne virus in the genus *Flavivirus* of the family *Flaviviridae*, is the etiologic agent of yellow fever, a viral hemorrhagic fever that occurs in South American countries and much of sub-Saharan Africa (*1**,**2*). Flaviviruses are single-stranded, positive-sense RNA viruses with a genome organization of 5´-C, prM, M, E, NS1, NS2A, NS2B, NS3, NS4A, 2K, NS4B, and NS5-3´ (*3**,**4*). YFV is transmitted by the bite of an infected female mosquito, usually *Aedes* species in Africa (*5*). YFV transmission has two epidemiologic patterns, the sylvatic (jungle) and urban cycles. The primary transmission cycle (sylvatic) involves nonhuman primates and tree hole–breeding mosquitoes. The urban cycle is defined by human involvement; humans are exposed to infected mosquitoes, which leads to infection (*5*). YFV, considered one of the reemerging human infections, is an important public health problem with a case-fatality rate that can exceed 50% in symptomatic patients (*6*).

YFV has been successfully isolated from acute-phase serum and liver samples by injection of suckling mice and cell cultures (*7*), and recent infections can be confirmed serologically by demonstrating immunoglobulin (Ig) M antibody to YFV in serum samples with a specific IgM-capture enzyme-linked immunosorbent assay (MAC-ELISA) (*8**,**9*). Viral antigen can be detected in liver tissue by using immunohistochemistry techniques, and viral nucleic acid can be detected in serum or tissue samples collected during the acute phase of illness by reverse transcription–polymerase chain reaction (RT-PCR) (*10*).

A safe and effective YFV vaccine, 17D, has been available since 1937. However, as it is not used universally, probably because of cost or inefficient networks for vaccination, the disease continues to occur in Africa and South America (*1*). Periodic outbreaks of yellow fever in East Africa have been reported since 1940 (*11*). The largest outbreak, estimated at 300,000 cases, occurred from 1960 to 1962 in Ethiopia (*12*). In 1966, yellow fever reappeared in Arba-Minch, Ethiopia, east of Lake Abaya, in an area unaffected by the outbreak of 1960. During the 1966 outbreak, 2,200 cases with 450 deaths were reported (*13*). Seropositivity of 14% had been recorded in northern Kenya (*14**,**15*), yet no outbreaks of yellow fever were reported until 1992, when an epidemic emerged in northwest Kenya, with at least 54 cases and 29 deaths (*16**,**17*).

On May 5, 2003, Norwegian Church Aid reported to the World Health Organization (WHO) office for southern Sudan a suspected disease outbreak in Imatong, a mountain range located in the southern part of Eastern Equatoria, southern Sudan. The range extends northwards to the town of Torit at 3,000 m above sea level. The mountain is covered by tropical rain forest with rich flora and fauna. Though the civil war has had a negative effect on the ecosystem, the Lango people continue to live on the eastern slopes of this mountain range. The total population in Imatong is estimated to be 24,387 people (WHO southern Sudan, 2003, unpub. data). Their primary means of livelihood is agriculture, although they also keep some livestock. According to the report by WHO’s Early Warning and Response Network (EWARN), which was established in southern Sudan in 1999 to monitor disease outbreaks, and the Norwegian Church Aid, the disease has clinical signs and symptoms of a viral illness and has caused the deaths of seven adults >30 years of age. On the basis of clinical symptoms, a viral hemorrhagic fever was suspected. On May 12, 2003, a team led by WHO arrived in southern Sudan to investigate and verify the cause of the outbreak. We report on laboratory investigations performed and the identified causative agent.

## Materials and Methods

### Collecting Human Serum

A case definition was established as follows: an illness in a patient of any age with high fever, severe headache, neck and back pain, possibly accompanied by vomiting, abdominal pain, diarrhea, hematemesis, bloody diarrhea, jaundice, and epistaxis. A total of 28 serum samples were collected: 18 from patients with acute disease who met the working case definition, 8 from patients with possible disease with headache and joint pain (patients 010, 013, 018, 020, 021, 022, 025, and 027), and 2 from convalescent patients (patients 020 and 028). Patient details, including their locations, are shown in the Table. Blood was collected in sterile evacuated clot activator Vacutainer tubes. Thick and thin smears were made on microscope slides for 17 patients as indicated in the Table, and the serum samples were then separated in the admission facility and stored in a liquid nitrogen dry shipper for transportation to the WHO Collaborating Center for Arbovirus and Viral Hemorrhagic Fever Reference and Research (CCAVHFRR) Laboratory at the Kenya Medical Research Institute (KEMRI) in Nairobi, where they were frozen at –70°C before testing.

### Serologic Testing

All the serum samples were heat-inactivated at 56°C for 30 min and tested for antibodies specific to flaviviruses by MAC-ELISA (*8**,**9*). Briefly, the plates were coated overnight at 4°C with anti-human IgM capture antibody (Kirkegaard and Perry Laboratories, Gaithersburg, MD), the plates were washed, and aliquots of test sera (positive and negative controls) were added in separate wells at a dilution of 1:400. After a 1-h incubation, the plates were washed, and each serum and control sample was reacted with sucrose-acetone extracted yellow fever antigen and control antigen at optimal dilution. Positive samples were detected with a commercially available monoclonal antibody against flaviviruses (6B6C-1) conjugated with horseradish peroxidase (Jackson Immunoresearch Laboratories, West Grove, PA) according to the manufacturer’s protocol and 2,2´-azino-bis(3-ethylbenthiazoline-6-sulfonic acid) substrate (Kirkegaard and Perry Laboratories). Absorbance was read at 405 nm. A presumptive diagnosis was made if IgM antibody in the test sample had a higher optical density than the ratio between the positive and negative control antigens.

### RT-PCR

All 28 serum samples were subjected to RT-PCR by using a universal pair of primers for members of the genus *Flavivirus*. Viral RNA was extracted directly from the samples by using the QIAamp viral RNA isolation kit (Qiagen, GmbH, Hilden, Germany). RT-PCR specific for amplification of a 267-bp region of NS5 gene of flaviviruses was performed with 50 pmol of oligonucleotide primers FU1 (TAC AAC ATG ATG GGA AAG AGA GAG AA) and CFD2 (GTG TCC CAG CCG GCG GTG TCA TCA GC) (*18*) and the Titan One Tube RT-PCR system (Roche Diagnostics GmbH, Mannheim, Germany), according to manufacturer’s instructions. The RT-PCR reactions were performed on a Perkin Elmer GeneAmp 9700 Thermocycler (Applied Biosystems, Warrington, England). The following cycling conditions were used: 50°C for 30 min followed by denaturation at 94°C for 3 min, 40 cycles of 94°C for 30 s, annealing at 55°C for 30 s, extension at 68°C for 30 s, and a final extension at 72°C for 7 min. PCR products were resolved by using a 1.5% agarose gel. A confirmatory RT-PCR was performed to amplify a 670-bp region of the YFV prM, M, and E genes with 50 pmol of oligonucleotide primers CAG (CTGTCCCAATCTCAGTCC) and YF7 (AATGCTTCCTTTCCCAAAT) (*19*). The following cycling conditions were used: 50°C for 30 min followed by denaturation at 94°C for 3 min, 40 cycles of 94°C for 30 s, annealing at 50°C for 30 s, extension at 68°C for 30 s, and a final extension at 72°C for 7 min. PCR products were then resolved on a 1.5% agarose gel. All the positive samples were exposed to a second RNA extraction and RT-PCR to rule out the possibility of contamination. Positive amplicons obtained with the *Flavivirus*-reactive primers were purified by excising the 267-bp fragment from the agarose gel and purifying the fragments with Qiagen gel purification kit (Qiagen) for nucleotide sequence determination.

### Nucleotide Sequencing

Gel-purified PCR products were quantitated, and the partial nucleotide sequences of amplicons with a minimum of 60 ng DNA/µL were determined by using Big Dye terminator sequencing ready reaction kits with AmpliTaq DNA polymerase FS (Applied Biosystems, Foster City, CA). A cycle sequencing reaction was performed with each of the primers CFD2 and FU1 in a final volume of 20 µL with 30 ng of PCR product, 3.5 pmol of primer, and 4 µL of BigDye Terminators premix, according to the manufacturer’s protocol. Briefly, the tubes were heated to 96°C for 2 min, and the reaction mixture underwent 25 cycles of 30 s at 96°C, 30 s at 50°C, and 4 min at 60°C. Excess Big Dye Terminators were removed by precipitation with absolute ethanol. The partial nucleotide sequence was obtained with an Applied Biosystems 377 sequencer, according to manufacturer’s instructions.

A search performed on Basic Local Alignment Search Tool (BLAST) (National Center for Biotechnology Information, U.S. National Library of Medicine, Bethesda, MD) suggested that the sequence obtained was amplified from the yellow fever genome. This finding was confirmed by aligning the sequence data with data from other known flaviviruses retrieved from GenBank.

### Virus Isolation

Aliquots of each of the serum samples were injected intracerebrally into litters of eight newborn (24–48 hours after birth) Swiss albino mice. The mice were observed daily for signs of illness. Sick mice were euthanized, and a preparation was made of 10% of the harvested mouse brain suspension made in Eagle’s Maintenance Media with 2% serum albumin, 2% glutamine, and 1% antibiotics (penicillin, streptomycin, and amphotericin B) and centrifuged at 3,000 rpm for 10 min. The clarified supernatant fluid was filtered with a 0.45-µm syringe filter and injected intracerebrally into a litter of suckling mice to confirm the isolation.

All the sera were diluted 1:10 in sterile phosphate-buffered saline pH 7.4 (without magnesium and calcium), and viral isolation attempts in Vero cell cultures were made by injecting 100 µL of the diluted sample onto confluent monolayer of Vero cells in 25-cm^2^ culture flasks. Flasks were incubated at 37°C and observed daily for evidence of cytopathogenic effect (CPE). After CPE was evident, the supernatant fluid was clarified by centrifugation at 3,000 rpm for 10 min. Viral RNA was extracted from both the 10% brain suspension from sick mice and from the clarified cell culture media and screened for flavivirus and YFV viral nucleic acid RNA by RT-PCR as described previously.

### Differential Diagnosis

IgM antibody tests were performed on all the samples to exclude recent exposure to Ebola virus, Marburg virus, Crimean-Congo hemorrhagic fever virus, West Nile virus, dengue virus, chikungunya virus, Sindbis virus, and Rift Valley fever virus. In addition, Ebola virus was excluded by using an antigen capture ELISA. PCR was performed to exclude the following pathogens: Crimean-Congo hemorrhagic fever virus, Rift Valley fever virus, Ebola virus, Marburg virus, rickettsia, leptospira, brucella, and members of the genus *Alphavirus* and *Bunyavirus*. For 17 patients, thick blood smears were checked for *Borrelia* spp. and thin blood smear slides were checked for malarial parasites.

## Results

On May 17, 2003, serologic tests and RT-PCR performed at the WHO CCAVHFRR at KEMRI identified YFV as the causative agent of the outbreak in southern Sudan. The diagnosis was supported by subsequent gene sequencing of the amplicons and isolation of the virus. The results obtained are summarized in the Table. Duplicate samples submitted to the Special Pathogens Unit at the National Institute for Communicable Diseases, South Africa, confirmed an outbreak of yellow fever.

### Detecting IgM Antibody to Flaviviruses

#### RT-PCR and Nucleotide Sequencing

Samples from five patients were positive for flavivirus viral nucleic acid and subsequently were positive for yellow fever viral nucleic acid with primers specific for YFV. Analysis of the nucleotide sequences obtained with the flavivirus primers confirmed that the amplicons were from the NS5 region of the yellow fever genome (phylogeny has been performed separately). The serum samples that were positive by PCR were collected from patients on day 1 after onset of illness except for one sample collected on day 8 from a patient with a fatal infection.

#### Viral Isolation

YFV was isolated, in both suckling mice and cell cultures, from five patients from whom PCR products were obtained. Virus isolation attempts on the remaining 23 serum samples from both suckling mice and Vero cell culture were negative.

### Differential Diagnosis

The sera were all negative by using various tests as described for Ebola virus, Marburg virus, Crimean-Congo hemorrhagic fever virus, West Nile virus, dengue virus, chikungunya virus, Sindbis virus, Rift Valley fever virus, rickettsia, leptospira, brucella, and members of the genus *Alphavirus* and *Bunyavirus*. The thick smears were negative for *Borrelia* by Giemsa staining. However, malaria parasites were demonstrated in thin smears from 7 of 17 patients tested; 6 of the patients with malaria also had evidence of YFV infection.

## Discussion

Samples from 28 patients were collected from various villages in the Imatong region as follows: 10 were from Locomo village, 3 from Lofi, 4 from Tarafafa, 3 from Imatong, and 1 each from Itohom, Lofulung, Ogolok, Lotodo, Lofong, and Itede; 2 were from unknown locations. A total of 18 patients had possible YFV infections demonstrated by detection of IgM antibody, while 5 patients were confirmed as positive for YFV by RT-PCR and virus isolation. Antibody was not demonstrable in the five patients who had positive RT-PCR results; in 4 patients, the specimens were collected too early (day 1) for antibody to be detectable. In patient 001 (Table), the specimen was collected on day 8 from a patient with a fatal infection. The patient was comatose and in the late stages of infection; antibodies were not found, possibly because of a failure to produce antibodies for a variety of factors, such as nutritional status, age, and immune status. This finding demonstrates the importance of using both modalities in the diagnosis of acute YFV infection.

**Table:** History, location and results obtained from patients during yellow fever outbreak, Imatong, southern Sudan, May 2003^a,b^

Case no.	Village	Sex	Age (y)	Days after onset	Flavivirus IgM	RT-PCR^a^	Virus isolation mice/cells	Blood smear MPS^c^
001^d^	Lofi	Male	48	8	–	+	+/+	nd
002^e^	Locomo	Male	60	4	–	–	nd^e^/–	–
003^e^	Tarafafa	Female	unknown	1	–	+	+/+	+
004^e^	Itohom	Female	12	Unknown	+	–	nd/–	+
005^e^	Tarafafa	Female	16	6	+	–	nd/–	+
006^e^	Locomo	Female	7	1	–	+	+/+	nd
007^e^	unknown	Male	unknown	Unknown	+	–	nd/–	+
008^e^	Lofi	Female	40	1	–	+	+/+	–
009^d^	Imatong	Female	35	Unknown	–	–	nd/–	nd
010^e^	Locomo	Female	15	Unknown	+	–	nd/–	+
011^e^	Locomo	Male	45	Unknown	+	–	nd/–	+
012^e^	Lofulang	Male	25	7	+	–	nd/–	nd
013^e^	Locomo	Female	8	1	–	+	+/+	nd
014^e^	Locomo	Male	60	Unknown	+	–	nd/–	–
015^e^	Ogolok	Male	42	Unknown	–	–	nd/–	–
016^e^	Locomo	Male	55	7	+	–	nd/–	–
017^e^	Tarafafa	Female	9	3	–	–	nd/–	–
018^e^	Lotodo	Female	3	Unknown	–	–	nd/–	–
019^d^	Imatong	Male	14	Unknown	–	–	nd/–	nd
020^f^	Imatong	Female	30	20	–	–	nd/–	+
021^f^	Lofi	Female	10	Unknown	+	–	nd/–	–
022^f^	Locomo	Male	25	Unknown	+	–	nd/–	nd
023^f^	Tarafafa	Male	60	Unknown	+	–	nd/–	nd
024^e^	unknown	Male	23	Unknown	–	–	nd/–	–
025^f^	Locomo	Female	40	Unknown	+	–	nd/–	nd
026^e^	Lofong	Female	16	Unknown	–	–	nd/–	–
027^e^	Locomo	Female	4	Unknown	+	–	nd/–	nd
028^f^	Itede	Female	23	21	–	–	nd/–	nd

^a^Ig, immunoglobulin; RT-PCR, reverse transcriptase-polymerase chain reaction; MPS, mucopolysaccharoidosis; nd, not done. ^b^Specimens were tested using primers reactive with genus *Flavivirus* and with a second set of primers specific to yellow fever virus. Nucleotide sequences were also determined. ^c^Malaria parasite smear. ^d^Fatal case. ^e^No outcome for cases due to inaccessibility. ^f^Recovered.

*Plasmodium*, the causative agent of malaria, was demonstrated in six patients who were positive for YFV. This finding demonstrates that co-infection with malaria and YFV is possible. Therefore, malaria should not be assumed as the etiologic agent of a disease outbreak solely based on positive screens, without first excluding other causes.

YFV maybe be enzootic in the Imatong Mountains, yet outbreaks have not been previously recorded in this region. Political unrest and population movement of highly susceptible (unvaccinated) people within the Imatong Mountains might have been a predisposing factor for the YFV outbreak in this region. During the civil unrest, large populations of monkeys were reportedly seen. As they became more dependent on human crops for food, the monkeys came closer to human habitations. Weather factors might have also contributed to the timing of the outbreak. The months of March to May 2003 were the rainy season, and heavy rains were reported around the mountain region. The persistent downpours may have resulted in high vector populations. With no history of human yellow fever in the outbreak zone, the monkey population must be adequate to support continued circulation of the virus in the sylvatic cycle. A combination of these factors likely contributed to the timing of the outbreak.

The early detection of this outbreak was due to EWARN, a WHO-facilitated network that brings together several healthcare providers and laboratories. One of the objectives of EWARN is the early detection and response to outbreaks, such as this one. The rapid response to this outbreak of YFV and subsequent laboratory-based diagnosis clearly demonstrate that an efficient surveillance system can lead to quickly detecting outbreaks and appropriate intervention, potentially saving many lives. At the time of this writing, 80% of the population at risk in Imatong had been vaccinated, with the campaign extending to Torit County and neighboring counties. Vaccination in Torit County had covered 51% of the population at risk (98,705).
